# ENLIST 1: An International Multi-centre Cross-sectional Study of the Clinical Features of Erythema Nodosum Leprosum

**DOI:** 10.1371/journal.pntd.0004065

**Published:** 2015-09-09

**Authors:** Stephen L. Walker, Marivic Balagon, Joydeepa Darlong, Shimelis N. Doni, Deanna A. Hagge, Vikas Halwai, Annamma John, Saba M. Lambert, Armi Maghanoy, Jose A. C. Nery, Kapil D. Neupane, Peter G. Nicholls, Vivek V. Pai, Pawan Parajuli, Anna M. Sales, Euzenir Sarno, Mahesh Shah, Digafe Tsegaye, Diana N. J. Lockwood, Erythema Nodosum Leprosum International STudy Group

**Affiliations:** 1 Faculty of Infectious and Tropical Diseases, London School of Hygiene and Tropical Medicine, London, United Kingdom; 2 Leonard Wood Memorial Center for TB and Leprosy Research, Cebu, Philippines; 3 The Leprosy Mission Hospital, Purulia, India; 4 ALERT Center, Addis Ababa, Ethiopia; 5 Anandaban Hospital, Kathmandu, Nepal; 6 Bombay Leprosy Project, Mumbai, India; 7 The Leprosy Mission Trust India, New Delhi, India; 8 FIOCRUZ, Rio de Janeiro, Brazil; 9 University of Southampton, Southampton, United Kingdom; University of California San Diego School of Medicine, UNITED STATES

## Abstract

Erythema nodosum leprosum (ENL) is a severe multisystem immune mediated complication of borderline lepromatous leprosy and lepromatous leprosy. ENL is associated with skin lesions, neuritis, arthritis, dactylitis, eye inflammation, osteitis, orchitis, lymphadenitis and nephritis. The treatment of ENL requires immunosuppression, which is often required for prolonged periods of time and may lead to serious adverse effects. ENL and its treatment is associated with increased mortality and economic hardship. Improved, evidence-based treatments for ENL are needed; however, defining the severity of ENL and outcome measures for treatment studies is difficult because of the multiple organ systems involved. A cross-sectional study was performed, by the members of the Erythema Nodosum Leprosum International STudy (ENLIST) Group, of patients with ENL attending seven leprosy referral centres in Brazil, Ethiopia, India, Nepal, the Philippines and the United Kingdom. We systematically documented the clinical features and type of ENL, its severity and the drugs used to treat it. Patients with chronic ENL were more likely to be assessed as having severe ENL. Pain, the most frequent symptom, assessed using a semi-quantitative scale was significantly worse in individuals with “severe” ENL. Our findings will determine the items to be included in a severity scale of ENL which we are developing and validating. The study also provides data on the clinical features of ENL, which can be incorporated into a definition of ENL and used for outcome measures in treatment studies.

## Author Summary

Leprosy is caused by a bacterium and is curable with a combination of antibiotics known as multi-drug therapy, which patients take for six or 12 months. However, a significant proportion of leprosy patients experience severe inflammation in their skin, nerves and other organs known as erythema nodosum leprosum (ENL). ENL is a severe complication of leprosy which can occur before, during, or after completion of antibacterial treatment. ENL causes severe morbidity affecting not only the skin, but also bones, joints, eyes, nerves, testes and kidneys. ENL may be fatal and it has been shown that the adverse effects of corticosteroid treatment also contribute to mortality. An effective treatment for ENL is thalidomide but this is not available in many leprosy endemic countries or is severely constrained by availability, cost and justified concerns about teratogenicity. Thalidomide, despite its effectiveness, is rarely used as a first line agent. Other effective drugs need to be identified to reduce the burden of this condition. In order to establish which treatments will be effective in ENL it is necessary to have a clear picture of the clinical features of the disease and how these influence severity. This is the first systematic study of ENL to define these and will help in developing tools to facilitate treatment studies of this severe, debilitating complication of leprosy.

## Introduction

Leprosy reactions are a significant cause of morbidity. Erythema nodosum leprosum (ENL) is an immunological complication affecting individuals with lepromatous leprosy (LL)[1]. ENL affects approximately 50% of individuals with LL and 5–10% of borderline lepromatous (BL) leprosy patients [1, 2].

ENL is a multisystem disorder characterised by the occurrence of crops of tender skin lesions. The histology of ENL lesions classically shows an intense perivascular infiltrate of neutrophils throughout the dermis and subcutis [3]. Tissue oedema and vessels exhibiting fibrinoid necrosis may also be present. The pathophysiology of ENL has not been fully elucidated though immune complexes and cellular immunity mechanisms have been reported be involved. ENL has some features of an immune complex mediated disease. Direct immunofluorescence studies have demonstrated granular deposits of immunoglobulin and complement in the dermis in ENL lesions but not in those of uncomplicated LL disease [4]. There is evidence of T lymphocyte and macrophage activation and expression of mRNA for TNFα and IL12 in the skin [5].

ENL may occur before, during or after successful completion of multi-drug therapy (MDT). An individual may have a single acute episode of ENL while others develop chronic forms lasting many years [1, 6]. It is not clear why some individuals develop ENL and others do not although polymorphisms in *NOD2* [7], *IL6* [8] and *NRAMP1* [9] genes have been associated with increased susceptibility for developing ENL.

The inflammatory state of ENL causes significant morbidity and mortality if untreated. Patients are treated with corticosteroids and thalidomide which are used for prolonged periods of many months or years. Many patients require high doses of corticosteroids to control their disease which leads to complications and a significant number of deaths associated with long-term use of these drugs [6, 10]. Thalidomide is not available in many countries or is severely restricted because of the risk of teratogenicity and cost. Thalidomide is also associated with adverse effects such as somnolence, nausea, neurotoxicity, dizziness and thromboembolism which may limit its use. ENL is also associated with severe economic hardship[11].

The identification of other agents for controlling ENL and the development of a quantitative measure to assess the severity of ENL have been highlighted as priority research areas [12, 13]. Since the introduction of multi-drug therapy (MDT) there have been only four randomised controlled trials involving just 136 individuals and only three of these involved some form of allocation concealment[14–17].

An understanding of the clinical features of ENL is important in the accurate diagnosis and successful management of the condition. The clinical features are also important as meaningful items for a severity scale and to determine appropriate outcome measures for intervention studies. Descriptions of leprosy reactions appear in many early monographs but the term ENL was first coined by Murata in 1912[18]. However there are no prospective studies detailing the clinical features and relative frequencies of organ involvement in ENL since the introduction of MDT. A retrospective study of the clinical features of ENL in 94 patients who had attended a “neuritis clinic” at a leprosy referral centre in Nepal were reported by Feuth et al[19]. This study reported that the most frequent non-cutaneous clinical signs in this cohort were: enlarged or tender nerves (99%), new and/or old nerve function impairment (NFI) (88%) and fever (78%).

A cross-sectional study was performed to gather data on the prevalence of the various clinical features of ENL including: the morphology of skin lesions, the presence of NFI and other extra-cutaneous manifestations. An assessment of the severity of pain associated with ENL was made. The severity and type of ENL (acute, recurrent or chronic) was recorded.

## Methods

The study was conducted in seven leprosy referral centres in Brazil, Ethiopia, India (two centres), Nepal, the Philippines and the United Kingdom by the ENLIST Group.

Leprosy was or had been diagnosed in individuals who had hypopigmented, anaesthetic skin patches and/or thickened nerves and/or acid-fast bacilli on slit skin smears. Leprosy was classified according to the classification of Ridley and Jopling[20] using clinical, histological and bacteriological indices.

The case definition of ENL was: “a patient diagnosed with leprosy has ENL if s/he has crops of tender cutaneous or subcutaneous lesions”. In the absence of cutaneous signs ENL could also be diagnosed if a patient with leprosy had fever or malaise and histological features consistent with ENL in a tissue biopsy.

The nature of ENL was defined as acute for a single episode lasting less than 24 weeks, recurrent if a patient experienced a second or subsequent episode of ENL occurring 28 days or more after stopping treatment for ENL and chronic if occurring for 24 weeks or more during which a patient has required ENL treatment either continuously or where any treatment free period had been 27 days or less[6].

Data were collected on individuals, at a single attendance at a participating centre, who were diagnosed with their first episode of ENL, a new episode of ENL in a patient previously diagnosed with ENL who was not receiving treatment for ENL or a deterioration of ENL while on ENL specific treatment. Patients with ENL on treatment who had no new or deteriorating symptoms, those diagnosed with a leprosy Type 1 reaction, those with Lucio’s phenomenon or a drug reaction at enrolment were excluded. Recruitment of the first individual occurred on 9^th^ May 2012 and the last on 30^th^ July2013. The period of recruitment at the non-UK centres ranged from 181–425 days. Patients underwent the usual clinical examination and investigations were performed only if that was the standard of care at the centre.

Using standardized definitions of symptoms and signs and a data collection form that had been agreed by all of the participating groups; demographic, clinical and laboratory data were recorded including evidence of any nerve function impairment (NFI) using voluntary muscle and Semmes-Weinstein monofilament sensory testing (which was the standard of care in all but one centre). New NFI was defined as NFI present for less than six months. Temperature measurements were taken from the axilla, oral cavity or tympanic membrane depending on the preference of each individual centre. Fever was defined as a temperature of greater than 37.5°C. The Wong-Baker Pain Rating Scale (range 0 “No pain” to 5 “Hurts worst”) [21] was used to assess pain. A physician determined assessment of ENL severity (mild or moderate or severe) was recorded. No attempt was made to standardise the severity assessments of the experienced study physicians prior to data collection because it was deemed beyond the scope of the study.

The anonymised data were entered into an Excel database and analysed using Stata 13 (StataCorp. 2013. *Stata Statistical Software*: *Release 13*. College Station, TX: StataCorp LP). The differences between groups was assessed using odds ratios, the Mann Whitney test, Chi squared test or ANOVA. The level for statistical significance was set at p ≤ 0.05.

### Ethics Statement

The study was routine. At all centres the data collected was that which pertained to the standard of care. At some centres no ethical approval was necessary. However Institutional Review Board approval was obtained for the centres in Brazil, Nepal and the Philippines. A mixture of written and verbal consent was obtained from the individuals studied.

## Results

Data were collected on 292 patients. The demographics, Ridley-Jopling classification of the patients’ leprosy, HIV status, type of ENL and physician determined severity of ENL are shown in Table 1.

**Table 1:** Demographic and clinical data of leprosy patients with ENL (n = 4 patients from UK not shown as a subgroup).

Centre		ALL (n = 292)	BRAZIL (n = 47)	ETHIOPIA (n = 51)	INDIA—BLP (n = 62)	INDIA—TLM (n = 60)	NEPAL (n = 47)	PHILIPPINES (n = 21)
**Male: Female**		**2.8:1 (215:77)**	3.7:1	1.1:1	3.8:1	5:1	3.3:1	20:1
**Median Age (years, [Range]):**		**32 [12–73]**	37 [19–73]	24 [12–55]	32 [14–68]	30 [12–65]	30 [18–60]	28 [21–50]
**Leprosy type**: **n (%)**	Borderline borderline (BB) leprosy	**5 (1.7)**	0 (0)	3 (5.9)	1 (1.6)	1 (1.7)	0 (0)	0 (0)
	BL leprosy	**65 (22.3)**	7 (14.9)	7 (13.7)	24 (38.7)	23 (38.3)	3 (6.4)	0 (0)
	LL	**222 (76.0)**	40 (85.1)	41(80.4)	37 (59.7)	36 (60.0)	44 (93.6)	21 (100)
**Median Mean Bacterial Index (BI) at leprosy diagnosis (288 BI available)**		**4 [0–6]**	4.5 [1.25–6]	3.5 [0–6]	4 [0.4–6]	4 [2–6]	4 [1–5.25]	5 [4.33–5]
**Reaction status at leprosy diagnosis: n (%)**	None	**113 (38.7)**	32 (68.1)	22 (43.1)	12 (19.4)	12 (20.0)	20 (42.6)	13 (61.9)
	Type 1	**13 (4.5)**	0 (0)	3 (5.9)	0 (0)	0 (0)	6 (12.8)	3 (14.2)
	ENL	**143 (49.0)**	13 (27.7)	26 (51.0)	49 (79.0)	35 (58.3)	19 (40.4)	1 (4.8)
	Neuritis	**23 (7.9)**	2(4.3)	0 (0)	1 (1.6)	13 (21.7)	2 (4.3)	4 (19.0)
**MDT status: (n = 291) n (%)**	No previous MDT	**17 (5.8)**	2 (4.3)	10 (19.6)	4 (6.5)	1 (1.7)	0 (0)	0 (0)
	Current	**101 (34.6)**	11 (23.4)	8 (15.7)	12 (15.4)	37 (61.7)	31 (66.0)	2 (9.5)
	Completed	**173 (59.2)**	34 (72.3)	33 (64.7)	45 (72.6)	22 (36.7)	16 (34.0)	19 (90.5)
**HIV status: n (%)**	Negative	**121 (41.4)**	22 (46.8)	49 (96.1)	---	---	47 (100)	——
	Positive	**1 (0.003)**	---	1 (2.0)	---	---	0 (0)	——
	Not known	**170 (58.2)**	25 (53.2)	1 (2.0)	62 (100)	60 (100)	0 (0)	21 (100)
**ENL type: n (%)**	Acute	**100 (34.2)**	17 (36.2)	21 (41.2)	21 (33.9)	24 (40.0)	9 (19.1)	8 (38.1)
	Recurrent	**95 (32.5)**	12 (25.5)	11 (21.6)	24 (38.7)	30 (50.0)	16 (34.0)	1 (4.8)
	Chronic	**97 (33.2)**	18 (38.3)	19 (37.3)	17 (27.4)	6 (10.0)	22 (46.8)	12 (57.1)
**Physician determined ENL severity (n = 289): n (%)**	Mild	**53 (18.3)**	10 (21.3)	3 (5.9)	21 (33.9)	2 (3.3)	16 (34.0)	1 (4.8)
	Moderate	**114 (39.4)**	14 (29.8)	16 (31.4)	31 (50.0)	49 (81.7)	2 (4.3)	0 (0)
	Severe	**122 (42.2)**	23 (48.9)	30 (58.8)	10 (16.1)	9 (15.0)	29 (61.7)	20 (95.2)

There were significant differences between centres in the study for the age of the patients (p = 0.001), Brazilian patients had the highest median age of 37 years and Ethiopian patients the lowest at 24 years. There were differences between centres with respect to Ridley-Jopling classification (p <0.001) although the majority of patients at each centre had LL. The proportion of patients with BL leprosy was considerably higher at the two Indian centres, at more than 38%, compared to the other centres.

There was an even distribution of acute, recurrent and chronic ENL in the study group. However a greater proportion of patients were deemed to have moderate or severe ENL than mild disease by the study physicians. There were significant differences between centres with respect to physician determined severity (p <0.001). A greater proportion of patients at the two Indian centres were categorised as having moderate ENL than elsewhere. Individuals with chronic ENL were more likely to be categorised as having severe disease than those with acute ENL [OR:2.75; 95% CI:1.54–4.91] or recurrent ENL [OR:4.29; 95% CI: 2.39–7.91].

The median duration of ENL at the time of enrolment in 106 of the 143 individuals who presented with ENL at the time of leprosy diagnosis was calculated and was 444 days [Range 0–6253]. The median duration of the episode of ENL studied in this study was seven days.

**Fig 1:**
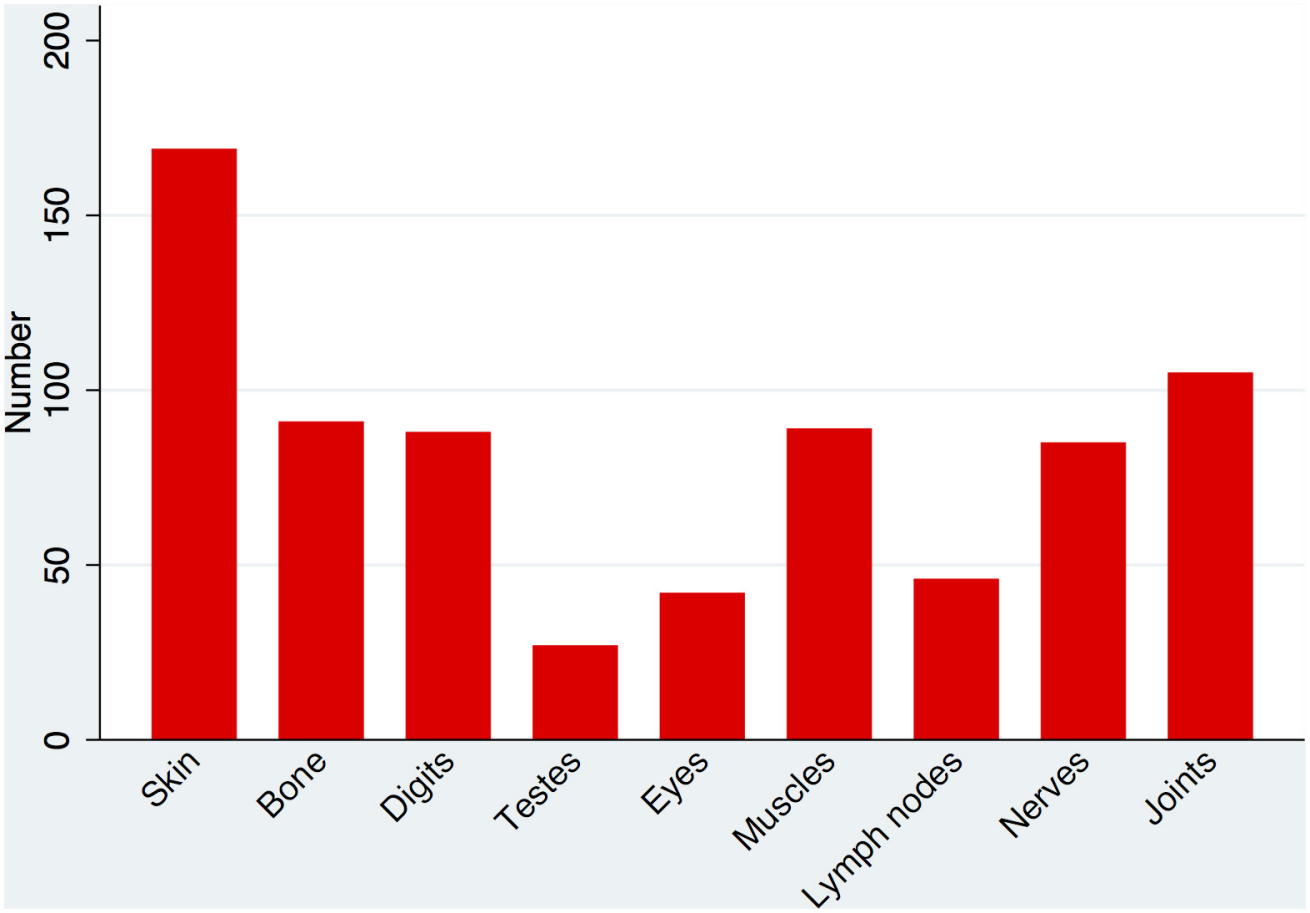
Location of pain in ENL.

**Fig 2:**
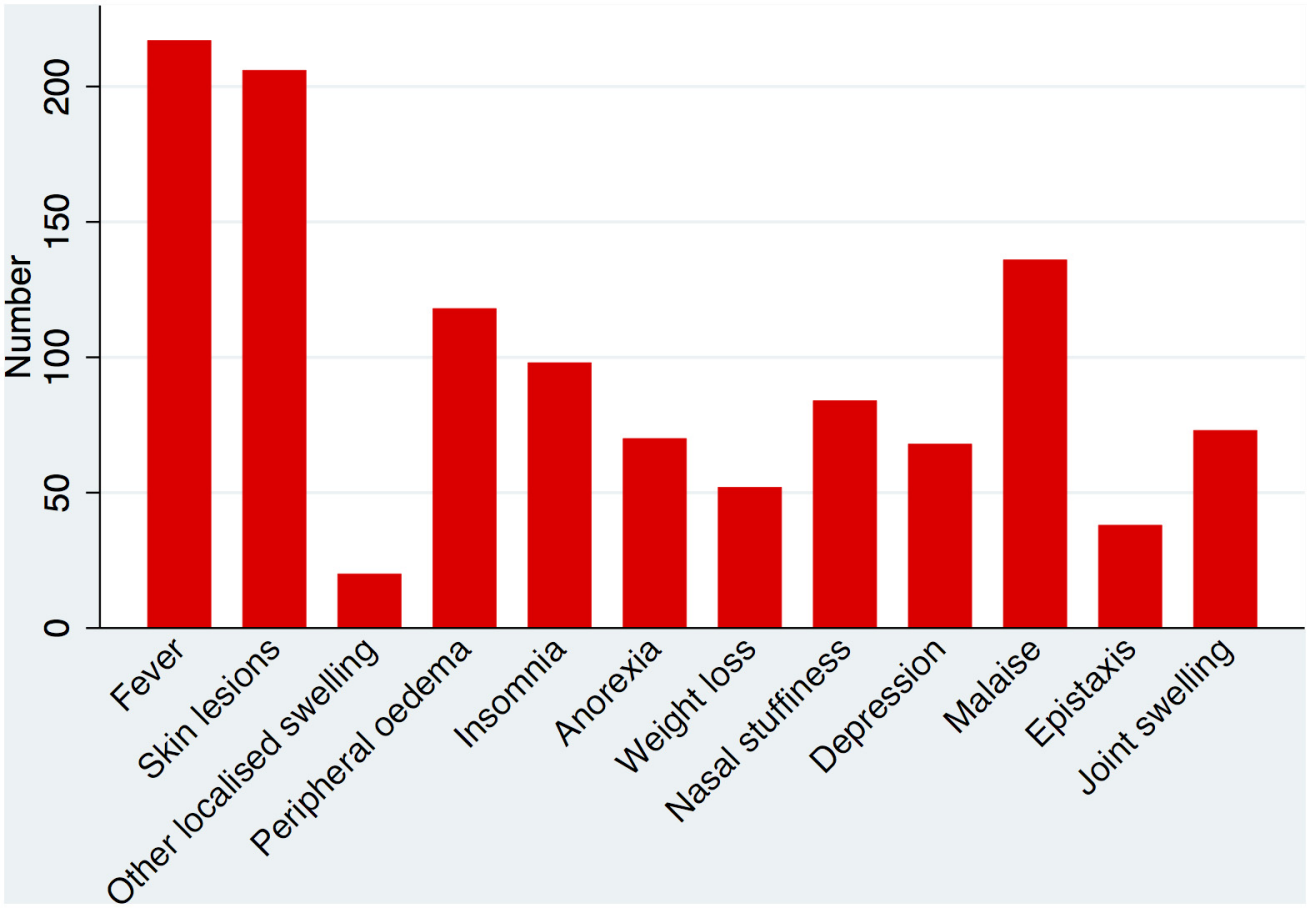
Symptoms other than pain in ENL.

Pain was the commonest symptom reported by patients. 279 (96.5%) ENL patients reporting some degree of pain. The location and frequency of the pain is demonstrated in Fig 1. The median number of symptoms reported by patients excluding pain was 4 [Range 0–12]. Fig 2 shows the distribution of the different symptoms. Patients with severe ENL were more likely to have more symptoms of ENL than those with mild or moderate ENL. Sixty-eight (23.3%) individuals complained of depression.

The median pain score using the Wong-Baker Pain Rating Scale was four (“hurts whole lot”) which is the second highest score possible. Of the 289 patients for whom pain data were collected, 169 (58.5%) reported pain intensity at one of the two highest possible levels (4 or 5).

The types of skin lesions and other organ involvement associated with ENL in the patients is shown in Table 2 for the group as a whole and the individuals centres.

**Table 2:** Clinical features of ENL (n = 4 patients from UK not shown as a subgroup).

Centre	ALL (n = 292)	BRAZIL (n = 47)	ETHIOPIA (n = 51)	INDIA—BLP (n = 62)	INDIA—TLM (n = 60)	NEPAL (n = 47)	PHILIPPINES (n = 21)
**Skin lesions**							
Nodules	**258**	43	43	42	60	47	20
Subcutaneous nodules	**69**	19	23	23	0	1	0
Papules	**52**	19	11	6	5	6	4
Plaques	**28**	12	7	4	0	5	0
Pustules	**29**	4	6	9	1	4	4
Vesicles	**12**	7	4	0	1	0	0
Bullae	**10**	2	3	0	1	3	0
Erythema multiforme-like	**13**	12	1	0	0	0	0
Necrotic	**11**	0	0	7	1	3	0
Ulcerated	**48**	1	16	8	12	8	3
**Other**							
Documented Fever	**56**	3	18	6	6	13	9
Oedema	**153**	20	35	41	36	13	5
NFI	**150**	29	21	30	36	18	14
Arthritis	**105**	11	22	31	19	14	6
Lymphadenitis	**43**	8	4	14	1	10	4
Dactylitis	**40**	5	11	11	5	7	0
Orchitis	**29**	7	2	5	1	8	6
Rhinitis	**23**	12	9	0	0	2	0
Ocular inflammation	**15**	0	1	4	5	3	1

**Fig 3:**
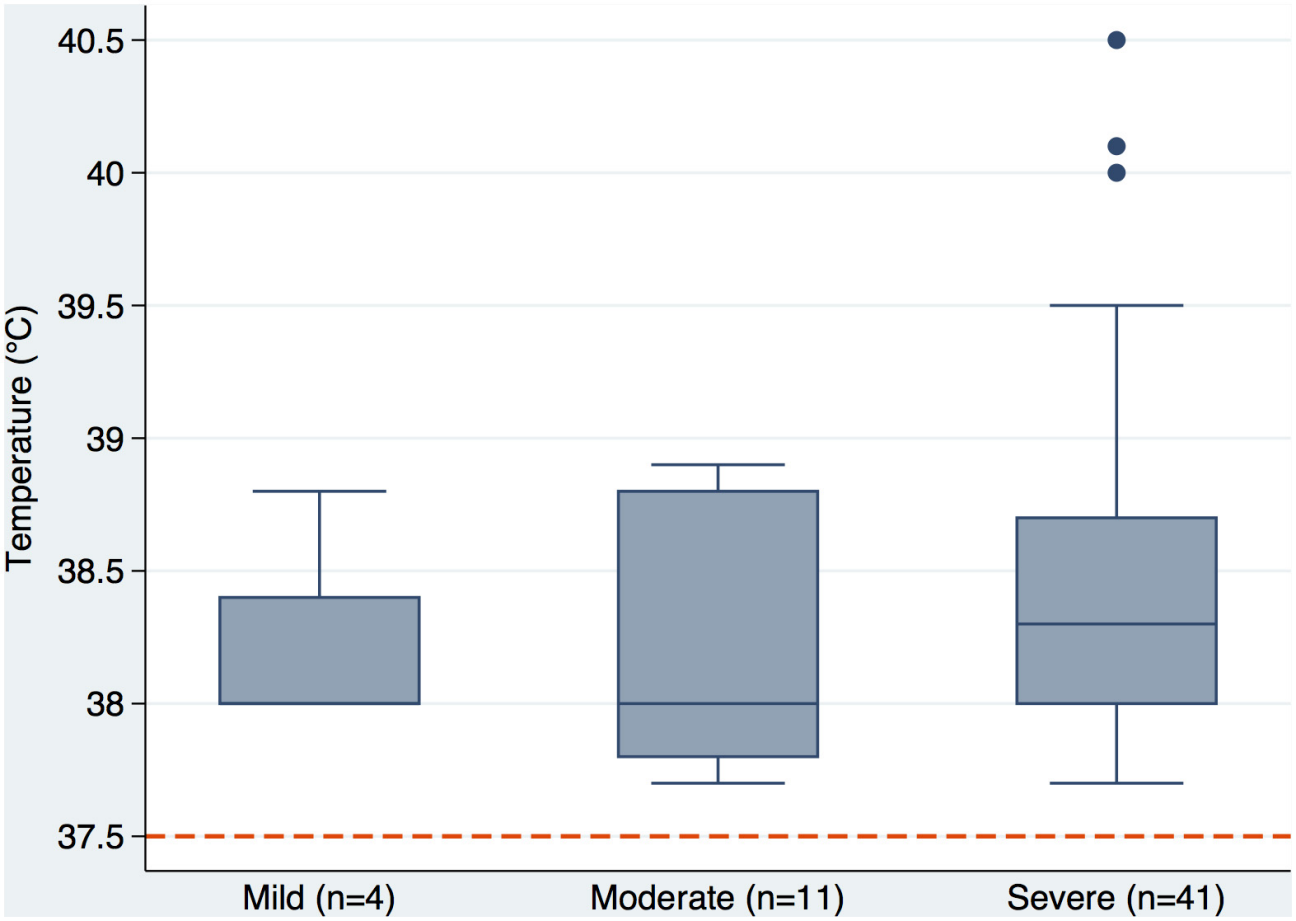
The degree of fever (temperature > 37.5°C) was not significantly different between groups with mild, moderate or severe ENL.

Fever was documented in 56 of 283 (19.8%) patients in contrast to the 217 (76.7%) who reported fever. The highest fever documented was 40.5°C. There was no significant difference in temperature between individuals with different types of ENL. However the group with severe ENL were significantly more likely to have a documented fever than those with mild ENL [OR:6.34; 95% CI: 2.13 to 18.84] or moderate ENL [OR:4. 95; 95% CI: 2.39 to 10.27]. There were no significant difference in the degree of fever exhibited by individuals in the different severity classifications of ENL Fig 3.

**Fig 4:**
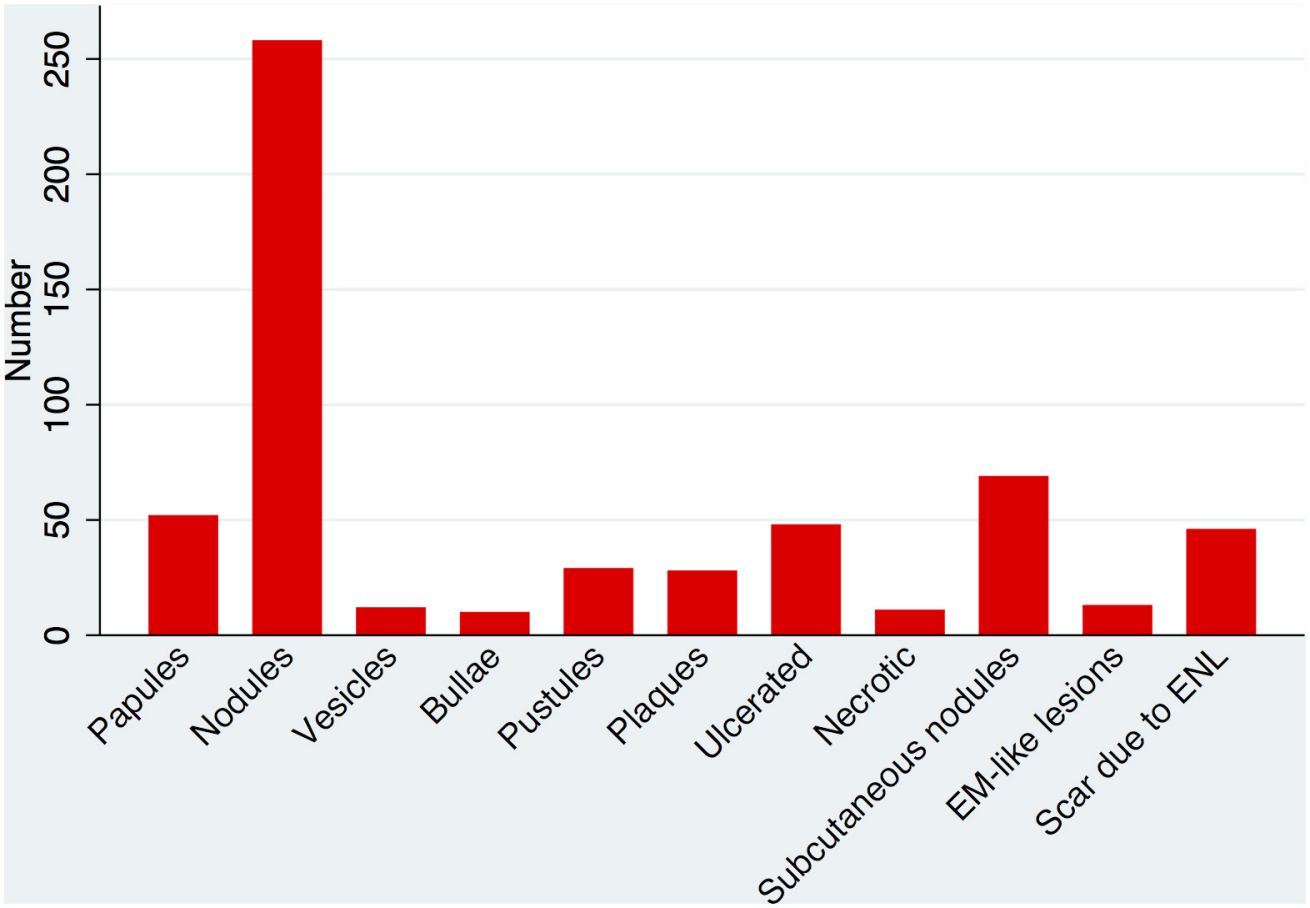
The frequency of the morphology of skin lesions in ENL (EM = erythema multiforme).

**Fig 5:**
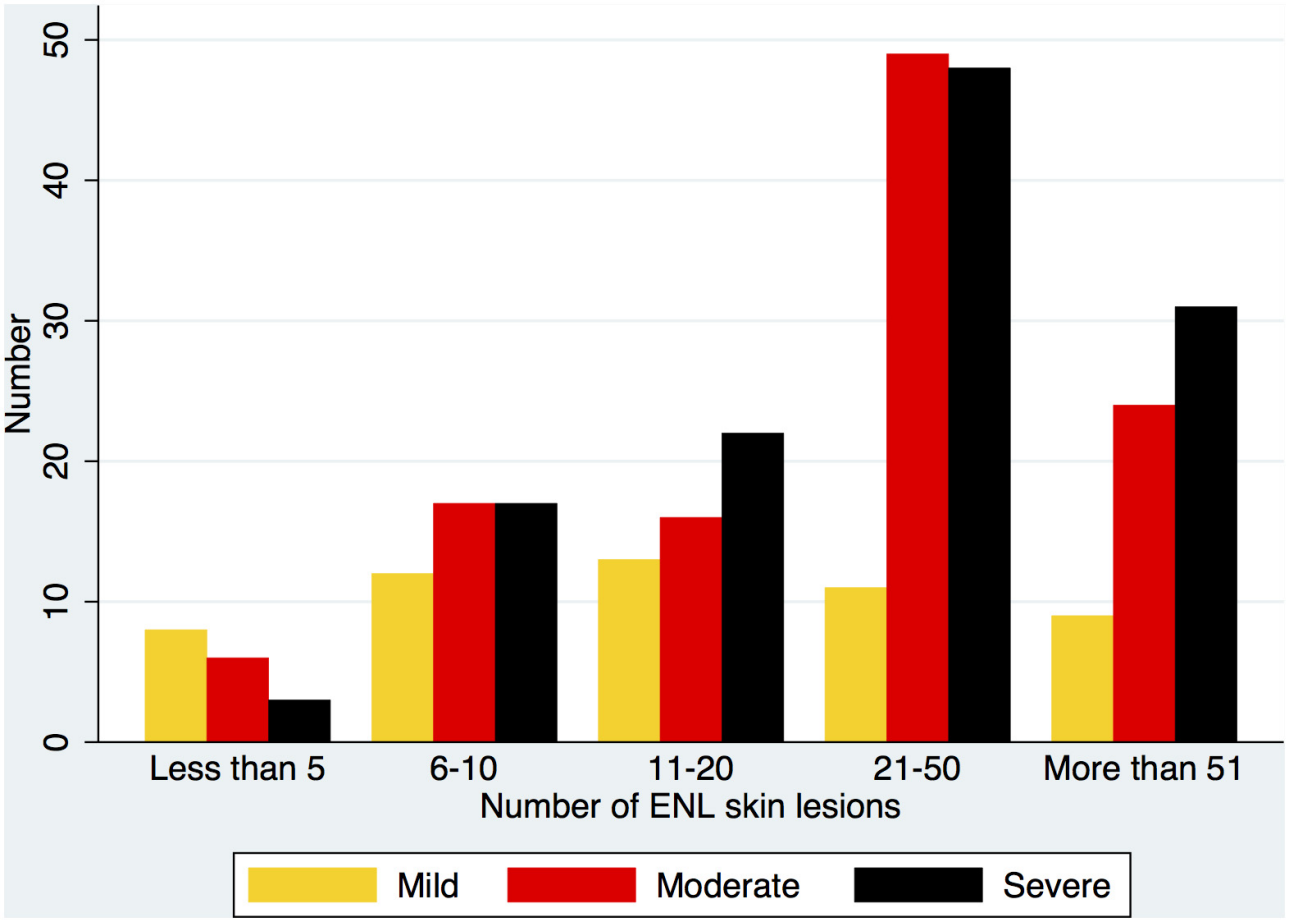
Number of skin lesions and severity of ENL.

The morphology of cutaneous lesions showed that 90.8% of individuals had papular or nodular lesions. 14% had vesicles, bullae or pustular lesions. Erythema multiforme-like lesions were documented in 4.4%. The frequency of the different types of skin lesion is illustrated in Fig 4. The size of the largest ENL lesion was not associated with physician determined severity although the number of individuals with ulcerated or necrotic skin lesions increased as ENL was judged to be more severe but these differences were not statistically significant. However the number of skin lesions and the severity of ENL were correlated. Individuals with more than 20 skin lesions were more likely to have moderate or severe ENL than those classified as having mild disease (p <0.001) (Fig 5).

**Fig 6:**
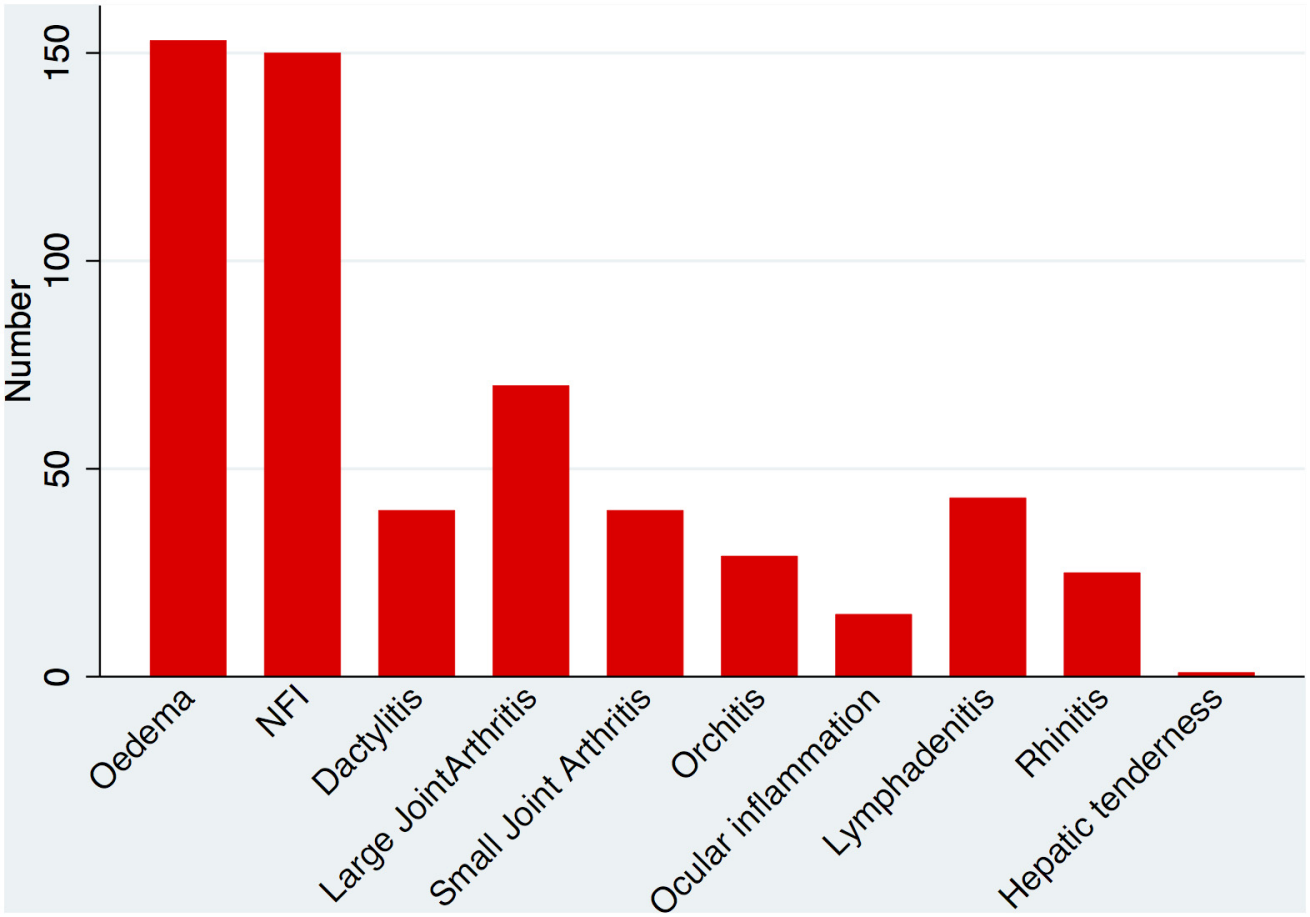
Organ involvement in ENL.

The examination findings also revealed significant amounts of extra-cutaneous pathology. Peripheral oedema was present in 153 (52.4%) of individuals, large joint arthritis in 70 (24%), lymphadenitis in 43 (14.7%) and orchitis was present in 29 of the 215 (13.5%) men. Other organ involvement is shown in Fig 6. Oedema was more often present at two or three sites (hands, feet or face) in those with more severe ENL.

**Fig 7:**
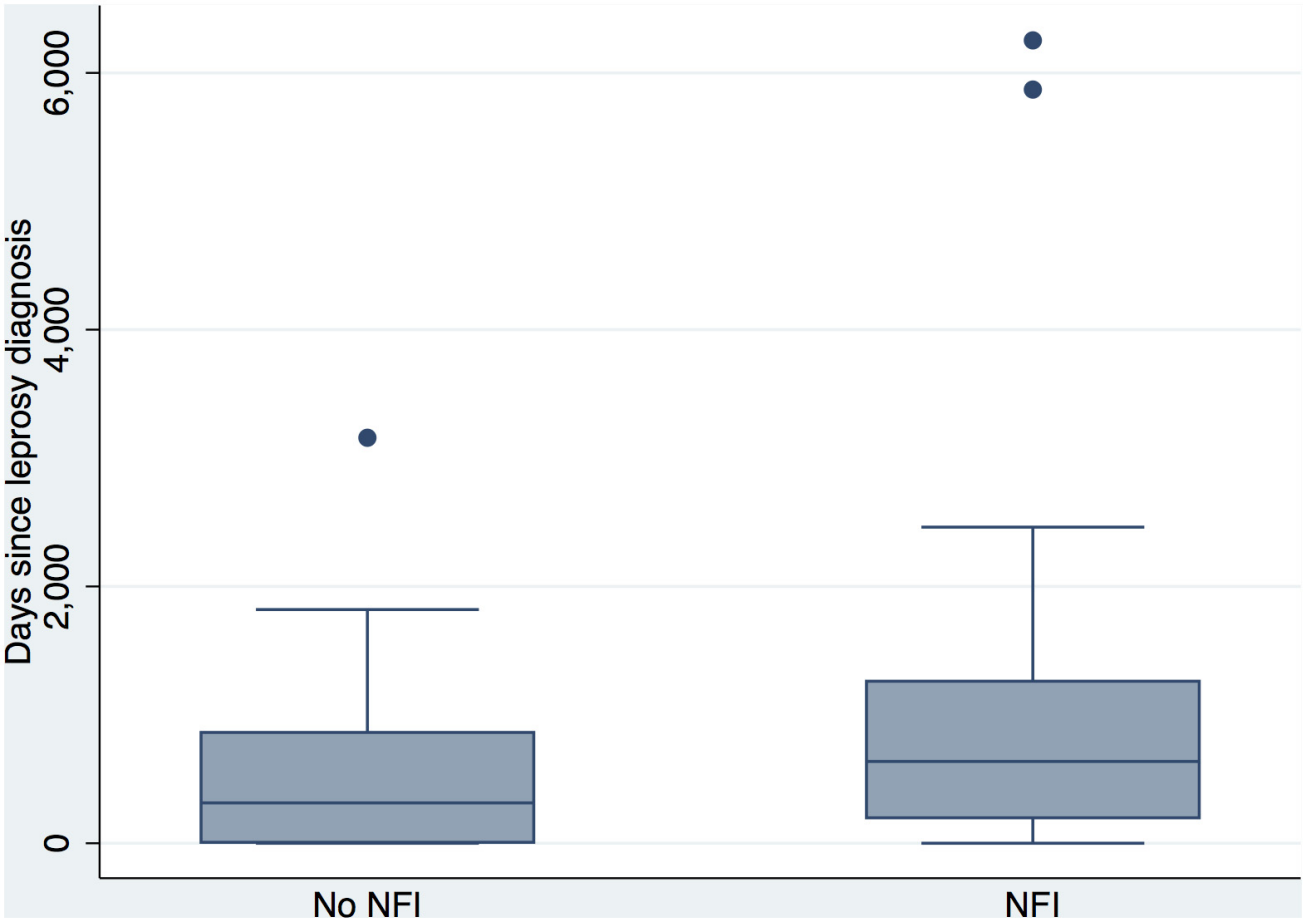
Duration of ENL and presence of nerve function impairment (NFI).

NFI was present in 150 (51.3%) individuals and of those 67 (22.9%) met the criteria for having new NFI. The presence of NFI was significantly associated with the duration of ENL in the 106 individuals who presented with ENL at the time of leprosy diagnosis see Fig 7 (p = 0.035).

Patients were prescribed a wide variety of treatments for their ENL. Data were available for 277 individuals. ENL treatment using one drug occurred in 193 (69.7%), two drugs in 73(26.4%) and three or more drugs in 11 (4%). Table 3 shows the ENL treatment prescribed at the study visit. The three most common treatments prescribed were prednisolone, prednisolone and clofazimine and thalidomide.

**Table 3:** ENL treatment prescribed for patients at the study assessment.

ENL treatment		ALL	BRAZIL	ETHIOPIA	INDIA—BLP	INDIA—TLM	NEPAL	PHILIPPINES	Type of ENL		
		Number (%) n = 277	(n = 47)	(n = 47)	(n = 60)	(n = 56)	(n = 47)	(n = 20)	ACUTE	RECURRENT	CHRONIC
**Monotherapy**	Prednisolone	**141 (50.9)**	2 (4.3)	28 (59.6)	23 (33.3)	40 (71.4)	28 (59.6)	20 (100)	**61**	**41**	**39**
	Clofazimine	**0 (0)**							**0**	**0**	**0**
	Thalidomide	**41 (14.8)**	37 (78.7)		3 (5.0)		1 (2.1)		**16**	**14**	**11**
	Pentoxifylline	**3 (1)**			3 (5.0)				**1**	**1**	**1**
	Non-steroidal anti-inflammatory drug (NSAID)	**8(2.9)**		1 (2.1)	7 (11.7)				**4**	**1**	**3**
**Two drugs**	Prednisolone and clofazimine	**55(19.9)**		18 (38.3)	17 (28.3)	14 (25.0)	6 (12.8)		**12**	**27**	**16**
	Prednisolone and thalidomide	**8(2.9)**	3 (6.4)		3 (5.0)	1 (1.8)	1 (2.1)		**1**	**2**	**5**
	Prednisolone and azathioprine	**3 (1)**					3 (6.4)		**0**	**0**	**3**
	Prednisolone and pentoxifylline	**6(2.2)**	4 (8.5)		2 (3.3)				**2**	**0**	**4**
	Prednisolone and NSAID	**1(0.4)**					1 (2.1)		**0**	**1**	**0**
**Three drugs or more**	Prednisolone, clofazimine and thalidomide	**5(1.8)**			2 (3.3)		3 (6.4)		**2**	**0**	**3**
	Prednisolone, clofazimine and azathioprine	**4(1.4)**					4 (8.5)		**0**	**1**	**3**
	Prednisolone, pentoxifylline and thalidomide	**1(0.4)**	1 (2.1)						**0**	**0**	**1**
	Prednisolone, clofazimine, thalidomide and NSAID	**1(0.4)**				1 (1.8)			**0**	**0**	**1**

## Discussion

This is the first systematic study of the clinical features of ENL and the profile of patients is similar to previous retrospective studies[1, 2, 19, 22]. The majority of patients are young, male and have lepromatous leprosy and a high mean BI. These data confirm that many individuals have chronic and severe disease. In this study 34.2% of individuals had acute ENL and a proportion of these will continue to experience ENL for longer than six months and therefore be reclassified as recurrent or chronic. Chronic ENL is significantly more likely to be classified severe than acute or recurrent ENL. This carries further weight when one considers that chronic ENL is probably the predominant type in referral centres where ENL is most likely to be managed[1, 6, 23].

Our study highlights the painful nature of ENL. Pain was reported by 96.5% of patients and was the most common symptom. Although the painful nature of ENL is recognised by clinicians this unpleasant feature of the disease is not prominently reflected in the literature. Using the semi-quantitative Wong-Baker Pain Rating Scale we were also able to show that not only is pain frequent but it is also severe.

In the retrospective study from Nepal[19] 76% of patients had joint pain, 52% nerve pain, 30% bone pain and 10% muscle pain although it is not clear how these were defined nor whether these symptoms were solely attributable to ENL. The most frequent site of pain due to ENL in our study was the skin which is not surprising given that skin lesions are a defining feature of the condition. The next commonest site for pain was the joints with 36.3% of individuals affected. Bone, muscle, digit and nerve pain were almost equally prevalent affecting 29.4–31.5% of individuals. The differences between these two studies are likely to be due to the methodological differences employed. The large number of people affected by severe pain suggests that patients experiencing ENL should have a formal pain assessment. Adequate analgesia in addition to immunomodulatory therapy is likely to be beneficial until control of the ENL has been established. Given the different organs that exhibit pain during ENL different analgesic regimes may be required. It was beyond the scope of this study to assess the type of pain experienced by patients with ENL but patients with leprosy experience different types of pain which require different interventions[24]

Fever is regarded as one of the hallmarks of ENL so we found it interesting that only 19.8% of patients had a documented fever on examination whilst 74.3% complained of fever. Feuth et al reported fever as a symptom in 78%. We conclude that a large proportion of patients experience fever or feel feverish due to the systemic inflammatory nature of ENL but that this does not necessarily result in actual pyrexia or the pyrexia may have subsided by the time they present. Although individuals classified as having severe ENL were more likely to be febrile than those with milder forms of ENL the degree of fever was not significantly different. These findings with respect to fever have implications for case definitions and severity assessments of ENL.

The cutaneous manifestations of ENL are more diverse than those in the case definition used in this study. The morphology of skin lesions other than papules and nodules is worthy of note in terms of the wider differential diagnoses that incorporate such lesions.

The number of inflammatory skin lesions appears to be related to severity and our study suggests the cut off is 20, a number that can be counted accurately.

The extracutaneous manifestations of ENL suggest that oedema is a common problem and a possible marker of severity. The prevalence of oedema was less than that described by Feuth et al but their methodology enabled them to capture data from multiple assessments of individual patients.

The high levels of NFI associated with ENL is significant (and in keeping with the data of Feuth et al from Nepal) because “neuritis” due to ENL is not well understood and thalidomide the gold standard treatment is not thought to be effective in the management of ENL associated neuritis and NFI. Most authorities advocate that this entity is treated with prednisolone. It is also important to recognise that we do not fully understand the potential neurotoxic adverse effects of thalidomide in individuals with ENL[10, 25].

The prevalence of cutaneous nodules, oedema and NFI as the three most common clinical signs in the cohort as a whole is reflected at the level of the individual centres. The two commonest clinical signs at each centre were either nodules and oedema or nodules and NFI. It would appear that ENL manifests in broadly the same way at the different centres, although this inference should be treated with caution due to the variety of potential confounding factors such as: ENL treatment, duration of ENL and MDT status.

The treatment of ENL at our centres varies considerably. Most patients were treated with prednisolone either alone or in combination with at least one other agent. Thalidomide is not available in Ethiopia or the Philippines. Its use is severely restricted in Nepal. In contrast patients are referred to the other centres for management of ENL with thalidomide. Although thalidomide is very effective in the management of ENL, 26.8% (15/56) patients in this study treated with the drug required at least one additional agent. One other striking feature about the treatment prescribed to these patients was that none of them received clofazimine monotherapy. Clofazimine was used in combination with prednisolone and its effectiveness as a steroid sparing agent should be properly assessed.

The limitations of this study were that, although data were collected systematically, we were only able to gather clinical data at one point in time. The study took place in referral centres in different countries and populations. We made no attempt to standardise the different physicians assessment of patients or ENL severity and we did not control for treatment that had been prescribed prior to the study assessment. The Wong-Baker Pain Rating Scale was developed in the USA for the assessment of children with pain and has not been validated in adults with ENL. It did not pose any problems of application in the different settings in which we used it. However these factors may account for some of the observed differences between centres. It is possible that there are genuine differences in ENL or the health-seeking behaviour of patients in these diverse settings and further work needs to consider these possibilities.

We have shown that ENL managed at leprosy referral centres is severe, very painful, associated with a high prevalence of neurological impairment and poses a considerable management problem even where thalidomide is available. This study provides the first detailed data of the prevalence of the clinical features of ENL and their relationship to physician determined severity that should be used to inform measures of ENL severity and outcome measures for much needed treatment studies.

## Supporting Information

S1 Checklist ENLIST 1 STROBE checklist. (DOCX)

S1 Database ENLIST 1 database. (XLSX)

## References

[1] Pocaterra L, Jain S, Reddy R, Muzaffarullah S, Torres O, Suneetha S, et al Clinical course of erythema nodosum leprosum: an 11-year cohort study in Hyderabad, India. Am J Trop Med Hyg. 2006;74(5):868–79. 16687695

[2] Kumar B, Dogra S, Kaur I Epidemiological characteristics of leprosy reactions: 15 years experience from north India. Int J Lepr Other Mycobact Dis. 2004;72(2):125–33. 15301592 10.1489/1544-581X(2004)072<0125:ECOLRY>2.0.CO;2

[3] Job CK. Pathology of leprosy In: Hastings RC, editor. Leprosy. 2nd ed Edinburgh: Churchill Livingstone; 1994 p. 193–234.

[4] Wemambu SN, Turk JL, Waters MF, Rees RJ. Erythema nodosum leprosum: a clinical manifestation of the arthus phenomenon. Lancet. 1969;2(7627):933–5. 4186599 10.1016/s0140-6736(69)90592-3

[5] Moraes MO, Sarno EN, Almeida AS, Saraiva BC, Nery JA, Martins RC, et al Cytokine mRNA expression in leprosy: a possible role for interferon-gamma and interleukin-12 in reactions (RR and ENL). Scand J Immunol. 1999;50(5):541–9. 10564558 10.1046/j.1365-3083.1999.00622.x

[6] Walker SL, Lebas E, Doni SN, Lockwood DN, Lambert SM. The mortality associated with erythema nodosum leprosum in Ethiopia: a retrospective hospital-based study. PLoS Negl Trop Dis. 2014;8(3):e2690 Epub 2014/03/15. 10.1371/journal.pntd.0002690 24625394 PMC3953021

[7] Berrington WR, Macdonald M, Khadge S, Sapkota BR, Janer M, Hagge DA, et al Common polymorphisms in the NOD2 gene region are associated with leprosy and its reactive states. J Infect Dis. 2010;201(9):1422–35. 10.1086/651559 20350193 PMC2853728

[8] Sousa AL, Fava VM, Sampaio LH, Martelli CM, Costa MB, Mira MT, et al Genetic and immunological evidence implicates interleukin 6 as a susceptibility gene for leprosy type 2 reaction. J Infect Dis. 2012;205(9):1417–24. Epub 2012/03/31. 10.1093/infdis/jis208 22459738

[9] Teixeira MA, Silva NL, Ramos Ade L, Hatagima A, Magalhaes V. [NRAMP1 gene polymorphisms in individuals with leprosy reactions attended at two reference centers in Recife, northeastern Brazil]. Rev Soc Bras Med Trop. 2010;43(3):281–6. 20563497

[10] Walker SL, Waters MF, Lockwood DN. The role of thalidomide in the management of erythema nodosum leprosum. Lepr Rev. 2007;78(3):197–215. 18035771

[11] Chandler DJ, Hansen KS, Mahato B, Darlong J, John A, Lockwood DN. Household costs of leprosy reactions (ENL) in rural India. PLoS Negl Trop Dis. 2015;9(1):e0003431 10.1371/journal.pntd.0003431 25590638 PMC4295874

[12] Van Veen NH, Lockwood DN, van Brakel WH, Ramirez J Jr., Richardus JH. Interventions for erythema nodosum leprosum. Cochrane Database Syst Rev. 2009;(3):CD006949 Epub 2009/07/10. 10.1002/14651858.CD006949.pub2 19588412 PMC11663503

[13] Walker SL, Saunderson P, Kahawita IP, Lockwood DN. International workshop on erythema nodosum leprosum (ENL)—consensus report; the formation of ENLIST, the ENL international study group. Lepr Rev. 2012;83(4):396–407. Epub 2013/04/26. 23614260

[14] Girdhar A, Chakma JK, Girdhar BK. Pulsed corticosteroid therapy in patients with chronic recurrent ENL: a pilot study. Indian J Lepr. 2002;74(3):233–6. 12708702

[15] Kaur I, Dogra S, Narang T, De D. Comparative efficacy of thalidomide and prednisolone in the treatment of moderate to severe erythema nodosum leprosum: a randomized study. Australas J Dermatol. 2009;50(3):181–5. Epub 2009/08/08. 10.1111/j.1440-0960.2009.00534.x 19659979

[16] Sales AM, Matos HJ, Nery JA, Duppre NC, Sampaio EP, Sarno EN. Double-blind trial of the efficacy of pentoxifylline vs thalidomide for the treatment of type II reaction in leprosy. Braz J Med Biol Res. 2007;40(2):243–8. 17273661 10.1590/s0100-879x2007000200011

[17] Villahermosa LG, Fajardo TT Jr., Abalos RM, Balagon MV, Tan EV, Cellona RV, et al A randomized, double-blind, double-dummy, controlled dose comparison of thalidomide for treatment of erythema nodosum leprosum. Am J Trop Med Hyg. 2005;72(5):518–26. 15891124

[18] Kikuchi I. Mosuke Murata, the designator of erythema nodosum leprosum. Lepr Rev. 2009;80(1):92–5. 19472858

[19] Feuth M, Brandsma JW, Faber WR, Bhattarai B, Feuth T, Anderson AM. Erythema nodosum leprosum in Nepal: a retrospective study of clinical features and response to treatment with prednisolone or thalidomide. Lepr Rev. 2008;79(3):254–69. 19009975

[20] Ridley DS, Jopling WH. Classification of Leprosy according to immunity. Int J Lepr Other Mycobact Dis 1966;34:255–73. 5950347

[21] Wong DL, Baker CM. Pain in children: comparison of assessment scales. Pediatric nursing. 1988;14(1):9–17. 3344163

[22] Nery JA, Vieira LM, de Matos HJ, Gallo ME, Sarno EN. Reactional states in multibacillary Hansen disease patients during multidrug therapy. Rev Inst Med Trop Sao Paulo. 1998;40(6):363–70. 10436656 10.1590/s0036-46651998000600005

[23] WHO. WHO Expert Committee on Leprosy 8th Report2012.

[24] Thakur S, Dworkin RH, Haroun OM, Lockwood DN, Rice AS. Acute and chronic pain associated with leprosy. Pain. 2015 10.1097/j.pain.0000000000000178 25830927

[25] Bastuji-Garin S, Ochonisky S, Bouche P, Gherardi RK, Duguet C, Djerradine Z, et al Incidence and risk factors for thalidomide neuropathy: a prospective study of 135 dermatologic patients. J Invest Dermatol. 2002;119(5):1020–6. 12445187 10.1046/j.1523-1747.2002.19502.x

